# Functional Outcomes and Gait Analysis of Total Hip Arthroplasty Through Lateral Hardinge Approach and Gluteus Medius-Sparing Approach: A Prospective Study

**DOI:** 10.7759/cureus.54323

**Published:** 2024-02-16

**Authors:** Narendra S Kushwaha, Ashish Kumar, Rohit Shukla, Dharmendra Kumar, Ganesh Yadav, Vineet Sharma, Mohammad Z Abbas, Kishore Parihar

**Affiliations:** 1 Department of Orthopaedics, King George’s Medical University, Lucknow, IND; 2 Department of Physical Medicine and Rehabilitation, King George’s Medical University, Lucknow, IND

**Keywords:** clinical outcome, harris hip score, gluteus medius sparing, hardinge approach, total hip arthroplasty

## Abstract

Background and objective

Hip degenerative joint disease is a common and debilitating musculoskeletal disorder. Total hip arthroplasty (THA) is a reconstructive hip procedure to relieve this condition through various surgical approaches. This study aimed to compare the functional outcomes between patients undergoing THA using the lateral Hardinge approach and the lateral gluteus medius-sparing approach.

Material and methods

This prospective study was carried out at a tertiary care institution. Thirty patients with arthritic hip joints were managed with total hip replacement (THR). The patients were allocated into two treatment groups; in group A, 14 patients received a THR by the lateral Hardinge approach, whereas in group B, 16 patients were managed by the lateral gluteus medius-sparing approach. Functional outcomes were assessed by the Harris Hip Score (HHS), and gait analysis was performed.

Results

The mean age of group A was 39.79 ±14.01 years and that of group B was 37.00 ±14.81 years. The mean length of incision was significantly lower in group B (p=0.001), whereas the mean duration of surgery (p=0.018) and mean contralateral pelvic tilt were found to be significantly lower in group A (p=0.009). No significant difference was found in abductor muscle strength, limb length discrepancy, HHS, pelvic obliquity, and pelvic rotation.

Conclusion

While functional outcomes were similar in both groups, the group that underwent THA with the gluteus medius-sparing approach had better gait based on lower pelvic tilt.

## Introduction

Coxarthrosis is one of the most common musculoskeletal diseases, and its prevalence is on the rise. Total hip arthroplasty (THA) is commonly performed to manage these conditions, and the major advantages of THA over conservative methods include better pain relief, improved outcomes, and cost-effectiveness [1]. The posterior approach of THA provides adequate visualization of the hip joint with easy accessibility for implant placement, especially for young surgeons, thereby making it one of the oldest and most widely used techniques with an established rehabilitation protocol. However, due to gluteus medius dissection, this approach has a potentially higher risk of early postoperative dislocation, and relatively more blood loss [2]. The anterior approach offers the advantage of significantly less muscle dissection and hence has a lower risk of dislocation with quicker rehabilitation. However, it is less commonly used due to limited visibility and implant accessibility and is associated with a steep learning curve [3]. The selection of the surgical approach should be based on factors such as blood flow, placement of the acetabular component, soft tissue integrity, hospital stay, and THA-associated complications such as nerve injury, hip abductor weakness, fracture, and hip dislocation, with their relative risks varying based on the chosen approach [1].

Hip replacement surgery requires good implant positioning as well as the optimum preservation of adjacent tissue to achieve good functional and clinical results. The lateral Hardinge approach ensures good access to the hip joint with a lower risk of dislocation but is associated with slower initial recovery and potential for limping or gait abnormality [4]. Our modification of the lateral Hardinge approach aims to maintain the anatomical integrity of the gluteus medius and provide good visualization of the surgical anatomy. The advantages of the modified gluteus medius-sparing lateral approach include complete visualization of the acetabulum, preservation of the femoral neck when a calcar femur-preserving (CFP) prosthesis is chosen, removal of osteophytes, corrosion protection of the acetabulum, and protection of the transverse ligament [5].

Improper functioning of the gluteus medius and decreased hip abduction force can lead to a limp and other associated issues such as the development of knee osteoarthritis [6] and pain [7] during stair climbing. Sparing the gluteus medius muscle during THA may alleviate this problem, thereby improving the quality of life for patients by reducing the dislocation rate associated with the standard anterolateral approach. Muscle deficiencies can cause modifications in gait in patients with hip abnormalities. To manage the decreased abductor force during the gait cycle, the patient's center of mass laterally shifts towards the top of the femur, resulting in a limp, a condition known as Trendelenburg gait [8]. This study was performed to compare the functional outcomes in patients who underwent THA using the lateral Hardinge approach and the lateral gluteus medius-sparing approach.

## Materials and methods

This study was carried out among patients attending the Department of Orthopaedic Surgery at a tertiary care center from August 2019 to July 2022. Institutional ethical clearance was obtained before subject enrolment (approval no: ECR/262/Inst/UP/2013/RR-16). Patients of either sex and aged 30-65 years with grade 3/4 arthritis of the hip joint and who were walking unsupported with normal other hip were enrolled and allocated to two treatment groups; group A consisted of patients who underwent THA by the lateral Hardinge approach, and group B comprised patients who received THA by the lateral gluteus medius-sparing approach. Patients who were obese (BMI >29.9 kg/m^2^), those with multiple comorbidities, and those needing revision surgery or hip replacement by other means were excluded from the study.

Surgical technique

Lateral Hardinge Approach

**Figure 1:**
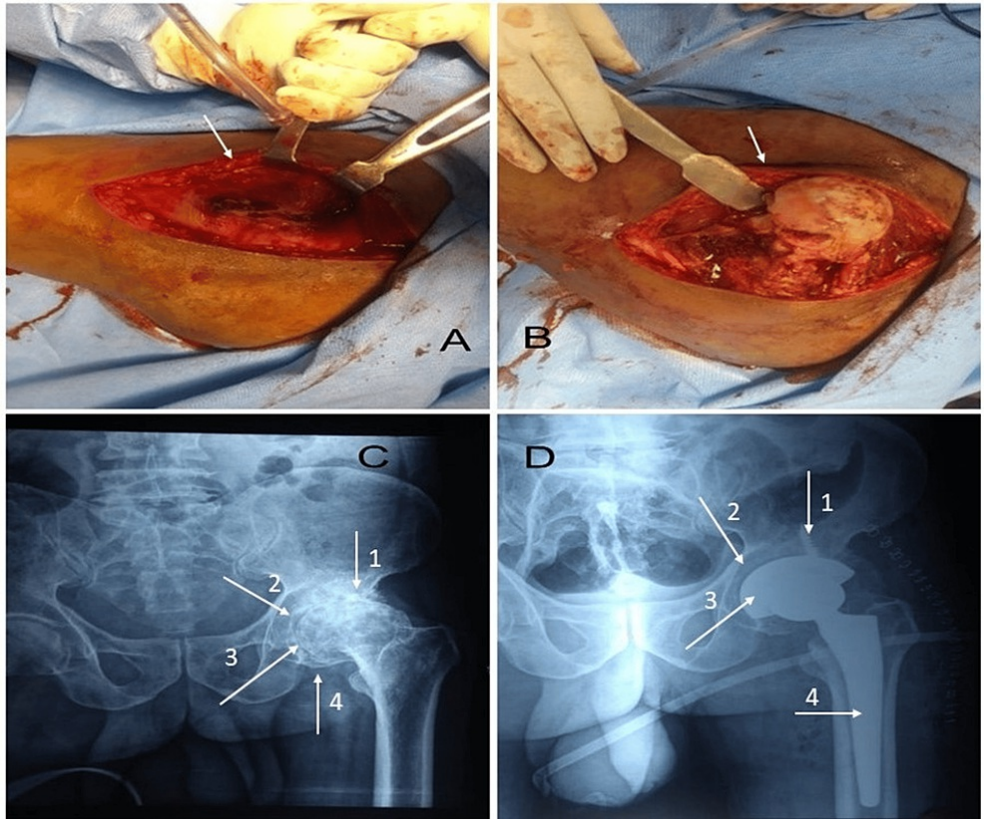
The lateral Hardinge approach A: the arrow shows the fascia split between the tensor fasciae latae (TFL) and gluteus maximus. B: the arrow shows the splitting of gluteus medius fibers at the anterior third and posterior two-third junction. C: radiographic image; arrow 1 shows subchondral sclerosis (looks more white ); arrow 2 demonstrates reduced joint space with a deformed femoral head; arrow 3 shows subchondral cyst; and arrow 4 shows osteophyte in the femoral head. D: radiographic image; arrow 1 shows the acetabular screw; arrow 2 shows the acetabular metal shell; arrow 3 points to the prosthetic femoral head; and arrow 4 shows the femoral stem

A curved incision in the shape of a posteriorly J was made 5 cm proximal to the tip of the greater trochanter, which passed over it and extended along the shaft of the femur for about 8 cm (Figure 1). After incising the fat and underlying deep fascia in line with the skin incision, tensor fascia lata and gluteus maximus fiber were retracted to visualize gluteus medius and vastus lateralis. Then, the anterior third of the gluteus medius and minimus was released from the greater trochanter while preserving the major attachment posteriorly. The anterior attachment of the hip capsule was next released from the anterior base of the femoral neck, and an anterior longitudinal capsulotomy was performed. After the prosthesis implantation, the gluteus minimus was sutured together, followed by the reapproximation of the combined insertion of the gluteus medius and vastus lateralis to the posterior tendinous cuff (Figure 1).

Lateral Gluteus Medius-Sparing Approach

**Figure 2:**
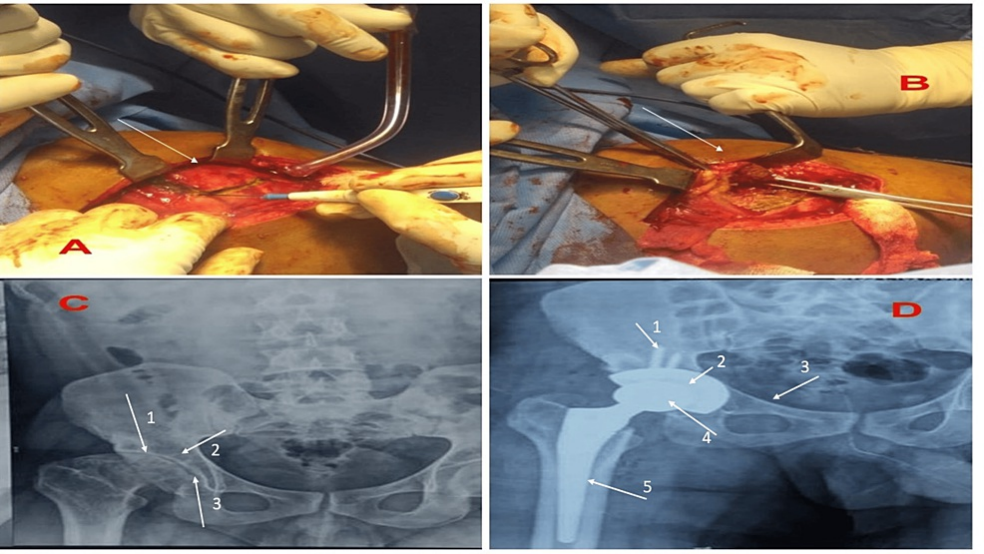
The lateral gluteus medius-sparing approach A: the arrow shows the incised anterolateral fibers of the vastus lateralis with fibers of the gluteus medius. B: the arrow shows the raised gluteus medius flap. C: radiographic images; arrow 1 shows subchondral cyst and sclerosis; arrow 2 shows the reduced joint space with a deformed femoral head; arrow 3 shows osteophytes in the femoral head. D: radiographic images; arrow 1 shows the acetabular screw; arrow 2 shows the acetabular metal shell; arrow 3 shows superior pubic rami; arrow 4 shows the prosthetic femoral head; and arrow 5 shows the femoral stem

With the patient in a strictly lateral position, a similar skin incision was made as in the Hardinge approach. The dissection was carried down and the fascia was incised in line with the skin incision. The plane between the tensor fascia latae (TFL) muscle anteriorly and the gluteus medius muscle posteriorly was bluntly developed using finger dissection. Blunt dissection was continued and the reflected head of the rectus femoris muscle was detached medially from the capsule. The capsule was then incised parallel to the neck extending from the inter-trochanteric line and up towards the acetabulum. After completing the capsulotomy, the acetabulum was prepared for the implant, and the acetabular component was inserted. Following the placement of the femoral prosthesis, the gluteus minimus was brought back together by using a strong absorbable suture, followed by the reapproximation of the combined insertion of the gluteus medius and vastus lateralis to the rear tendinous cuff. All patients who met the specified inclusion criteria and expressed willingness to participate in the study were evaluated for factors such as age, gender, preoperative range of motion (ROM) and its measurement at three and six months, the Harris Hip Score (HHS) both preoperatively and at three and six months, parameters of gait analysis (pelvic tilt, pelvic rotation, pelvic and obliquity), as well as the duration of the surgical procedure (Figure 2).

Gait analysis protocol

Every participant in the study was made to walk without footwear along a designated 11-meter pathway within the gait and motion laboratory, adhering to the established WALK protocol outlined by the BTS G-WALK system (BTS Bioengineering USA, Quincy, MA). The walking was conducted at a pace that was natural and self-selected by each individual. The BTS G-WALK system was employed to facilitate motion analysis, capturing various spatiotemporal parameters related to the participants' gait patterns. This approach enables a comprehensive examination of how different surgical approaches impact the way participants walk, providing insights into their walking patterns and timing.

Statistical analysis

The data were analyzed using SPSS Statistics version 21.0 (IBM Corp., Armonk, NY). A p-value <0.05 was considered statistically significant.

## Results

The mean age of group A was 39.79 ±14.01 years and that of group B was 37.00 ±14.81 years, and the difference was statistically insignificant. Both the groups had comparable mean BMIs (group A: 26.7 ±0.21 kg/m^2^; group B: 26.3 ±0.18 kg/m^2^). The male-female proportion (p=0.91), and sites of injury on the left and right in group A (64.3%:35.7%) and group B (43.8%:56.3%) (p=0.261) were observed. The mean duration of surgery in group B (2.00 ±0.37 hours) was significantly longer than in group A (1.69 ±0.31 hours) (p=0.018), as shown in Table 1.

**Table 1:** Comparison of demographic and clinical data between the two groups SD: standard deviation

Variables	Group A	Group B	T-value	P-value
Number of subjects (%)	14 (46.7)	16 (53.3)	0.15	0.883
Age, years, mean ±SD	37.79 ±14.01	37.00 ±14.81		
Gender, n (%)	Male: 9 (64.3)	10 (62.5)		0.919
	Female: 5 (35.7)	6 (37.5)		
Site of surgery, n (%)	Right: 5 (35.7)	9 (56.3)	1.27	0.261
	Left: 9 (64.3)	7 (43.8)		
Length of incision, cm, mean ±SD	11.71 ±1.44	8.28 ±3.33	3.57	0.001
Duration of surgery, hours, mean ±SD	1.69 ±0.31	2.00 ±0.37	-2.51	0.018
Abductor muscle strength, mean ±SD	Preop: 3.29 ±1.49	3.56 ±1.26	-0.55	0.586
	3 months: 3.86 ±0.36	3.94 ±0.77	-0.36	0.725
	6 months: 3.93 ±0.47	3.94 ±0.77	-0.44	0.970
Limb length discrepancy, mean ±SD	Preop: 1.08 ±1.04	1.00 ±1.04	0.19	0.849
	3 months: 0.19 ±0.48	0.30 ±0.46	-0.61	0.548
	6 months: 0.19 ±0.48	0.23 ±0.42	-0.24	0.811
Harris Hip Score (HHS), mean ±SD	Preop: 45.74 ±12.11	43.69 ±7.72	0.56	0.580
	3 months: 67.71 ±9.76	66.30 ±8.31	0.43	0.671
	6 months: 73.22 ±7.26	71.39 ±8.01	0.65	0.519
Pelvic tilt, mean ±SD	Preop: 2.35 ±0.93	3.19 ±1.42	-1.88	0.071
	3 months: 3.28 ±1.49	2.70 ±1.10	1.22	0.231
	6 months: 3.76 ±1.26	2.49 ±1.21	2.81	0.009
Pelvic obliquity, mean ±SD	Preop: 6.01 ±1.75	6.06 ±1.99	-0.07	0.945
	3 months: 7.05 ±1.94	7.16 ±1.78	-0.16	0.877
	6 months: 7.78 ±1.79	6.88 ±1.82	1.36	0.185
Pelvic rotation, mean ±SD	Preop: 6.01 ±1.37	6.58 ±1.71	-1.00	0.324
	3 months: 7.76 ±2.28	7.02 ±2.55	0.84	0.409
	6 months: 7.16 ±2.92	6.16 ±1.89	1.12	0.272

**Figure 3:**
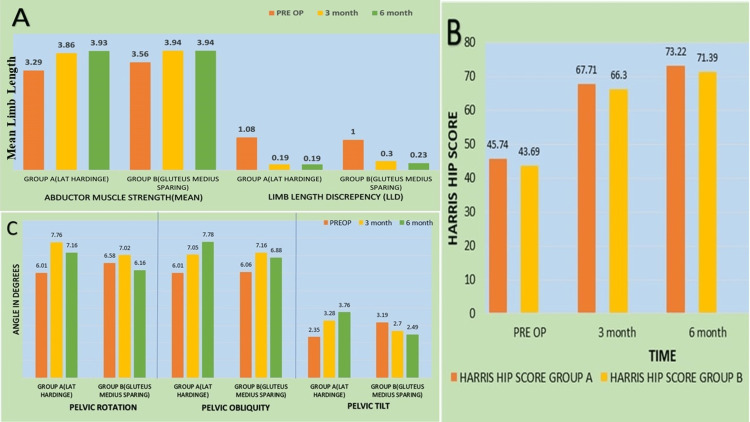
Gait analysis 3A: preoperative abductor muscle strength and limb length in both groups; 3B: Harris Hip Score; 3C: pelvic parameters during the study

While the difference in CPT between group A and B was not significant preoperatively (p=0.071) and after six months (p=0.231), it was found to be significantly lower in group B than in group A (p=0.009) at three months (Figure 3C). The difference in pelvic obliquity between groups A and B was not found to be significant preoperatively (p=0.945), after three months (p=0.877), and six months (p=0.185) (Figure 3C). The mean limb length discrepancy in group A was 1.08 ±1.04 preoperatively while it was 1.00 ± 1.04 in group B. The difference in the discrepancy between groups A and B was not found to be significant preoperatively (p=0.849), at three months (p=0.548), and at six months (p=0.811). The mean HHS in group A was 45.74 ±12.11 preoperatively while it was 43.69 ±7.72 in group B (Figure 3B). Preoperatively, the maximum proportion of ROM (abduction) in groups A and B was 0-20, which was statistically insignificant (p=0.405). A limp or deviation from the normal expected walking pattern may be attributed to pain, weakness, or a structural abnormality. The lateral Hardinge approach involves the dissection of the anterior part of the gluteus medius in relation to the greater trochanter and hence the chances of postop abductor weakness or limping are substantial if proper muscle repair is not ensured.

## Discussion

Our study demonstrated the clinical significance of sparing hip abductor dissection in the surgical approach of THA. A range of surgical methodologies is employed to execute THA, each characterized by its own unique set of advantages and disadvantages. The selection of a specific surgical approach can exert varying effects on hip functionality, primarily attributable to preservation factors [9]. Some authors compared the various surgical techniques in relation to the hip function [10-13]. In this study, we conducted a comparative analysis of functional outcomes after THA, specifically evaluating the outcomes of two distinct surgical approaches: the lateral gluteus medius-sparing approach and the lateral Hardinge approach.

Notably, both study groups were comparable in terms of age (p=0.31) and sex distribution (p=0.919). It is noteworthy that our study's age demographic differs from that of the study by Wayne and Stoewe, where their subjects had an average age of 68 years, in contrast to our study's average age of 37.39 years. However, the gender distribution in our study was similar to theirs [12]. The average surgical duration for the lateral gluteus medius-sparing approach turned out to be two hours due to challenges encountered during the window formation between the gluteus medius and TFL, whereas the lateral Hardinge approach required 1.69 hours. In terms of incision length, group A (managed via the lateral Hardinge approach) exhibited an incision of 11.71 cm, while group B (managed via the lateral gluteus medius-sparing approach) had a significantly smaller incision of 8.28 cm (p=0.001).

Both groups showed improvements in their HHS at the three- and six-month follow-ups. Patients in group A saw their scores increase from a mean of 45.53 to 60.91, and those in group B observed a change from a mean of 46.67 to 56.98. However, these changes did not reach statistical significance. Hence, both groups had a very low risk of revision risk post-primary THA. This aligns with the findings by Restrepo et al., who observed enhanced HHS at six weeks, three months, and six months postoperatively with the direct anterior approach when compared to the direct lateral approach [13]. Barrett et al. also reported improved HHS at six weeks postoperatively with the direct anterior approach over the posterior approach [14]. Barber et al., in a study involving a two-year follow-up in 28 patients undergoing direct posterior and 21 patients undergoing direct lateral THA, reported no significant difference in improvements in the HHS [15].

In our study, gait analysis was performed on each patient, evaluating contralateral pelvic tilt, pelvic obliquity, and pelvic rotation in both groups preoperatively, at three months, and six months postoperatively. Interestingly, there was no significant disparity between the two groups concerning contralateral pelvic tilt at the three-month follow-up (p=0.231). However, a noteworthy difference emerged at the six-month mark (p=0.009). These findings align with the findings of a study by Petis et al., which reported a significantly greater contralateral pelvic tilt in the lateral approach compared to the anterior approach at six weeks postoperatively (p=0.041), though this difference became insignificant at the six-month assessment [16]. The statistical disparities in anatomic factors resulting from different surgical approaches may indeed contribute to these findings. However, the clinical significance of these variations has been elucidated by Takacs et al., who have pointed out that as pelvic tilt increases, there is an associated increase in energy expenditure [17]. In our study, no statistically significant differences were detected in hip ROM between the two groups either during the preoperative period or at any of the follow-up assessments. This finding aligns with the results reported by Yoo JI et al., who also found no statistically significant differences in hip ROM in the sagittal plane within three months following THA between the two groups (p=0.24) [18].

This study has a few limitations. A comparative analysis of the temporal parameters of gait was not feasible. Furthermore, the single-center design we employed imposed constraints regarding the broader generalizability of the data, primarily because only two surgeons were involved in performing the procedures. Hence, prudence is warranted when extrapolating these findings to a wider population or considering the comprehensive spectrum of gait-related attributes.

## Conclusions

Based on our findings, the gluteus medius-sparing approach in THA shows promising benefits in improving gait patterns and reducing pelvic tilt during postoperative recovery. While this technique contributes positively to postoperative gait dynamics by preserving the integrity of the gluteus medius, functional outcomes, patient satisfaction, pain relief, and the restoration of daily activities appear comparable to conventional methods. Future research involving larger and more diverse participant cohorts, along with extended follow-up durations, is recommended to validate our findings about the advantages of the gluteus medius-sparing technique.
